# Economic burden of nosocomial infections caused by vancomycin-resistant enterococci

**DOI:** 10.1186/s13756-017-0291-z

**Published:** 2018-01-05

**Authors:** Laura Puchter, Iris Freya Chaberny, Frank Schwab, Ralf-Peter Vonberg, Franz-Christoph Bange, Ella Ebadi

**Affiliations:** 1 Department of Anesthesiology and Intensive Care Medicine, KRH Klinikum Hannover, Hannover, Germany; 2 Institute of Infection Control and Hospital Epidemiology, Leipzig University Hospital, Leipzig, Germany; 3 Institute of Hygiene and Environmental Medicine, Charité - University Medicine, Berlin, Germany; 4 Institute for Medical Microbiology and Hospital Epidemiology, Hannover Medical School, Carl-Neuberg-Straße 1, 30625 Hannover, Germany

**Keywords:** Attributable costs, Financial loss, Vancomycin-resistant *Enterococcus*, Nosocomial infection

## Abstract

**Background:**

Nosocomial infections due to vancomycin-resistant *enterococci* (VRE) have become a major problem during the last years. The purpose of this study was to investigate the economic burden of nosocomial VRE infections in a European university hospital.

**Methods:**

A retrospective matched case-control study was performed including patients who acquired nosocomial infection with either VRE or vancomycin-susceptible enterococci (VSE) within a time period of 3 years. 42 cases with VRE infections and 42 controls with VSE infections were matched for age, gender, admission and discharge within the same year, time at risk for infection, Charlson comorbidity index (±1), stay on intensive care units and non-intensive care units as well as for the type of infection, using criteria of the Centers for Disease Control and Prevention.

**Results:**

The median overall costs per case were significantly higher than for controls (EUR 57,675 vs. EUR 38,344; *p* = 0.030). Costs were similar between cases and controls before onset of infection (EUR 17,893 vs. EUR 16,600; *p* = 0.386), but higher after onset of infection (EUR 37,971 vs. EUR 23,025; *p* = 0.049). The median attributable costs per case for vancomycin-resistance were EUR 13,157 (*p* = 0.036). The most significant differences in costs between cases and controls turned out to be for pharmaceuticals (EUR 6030 vs. EUR 2801; *p* = 0.008) followed by nursing staff (EUR 8956 vs. EUR 4621; *p* = 0.032), medical products (EUR 3312 vs. EUR 1838; *p* = 0.020), and for assistant medical technicians (EUR 3766 vs. EUR 2474; *p* = 0.023). Furthermore, multivariate analysis revealed that costs were driven independently by vancomycin-resistance (1.4 fold; *p* = 0.034).

**Conclusions:**

This analysis suggested that nosocomial VRE infections significantly increases hospital costs compared with VSE infections. Therefore, hospital personal should implement control measures to prevent VRE transmission.

## Background

Although vancomycin-resistant *enterococci* (VRE) are organisms of low virulence and low pathogenicity, they frequently cause nosocomial infections [1, 2]. A significant increase in the VRE prevalence has been observed in many countries recently. For example the proportion of vancomycin-resistant *Enterococcus faecium* has increased rapidly from <5% in 2001 to 14.5% in 2013 in Germany [3]. In the USA surveillance data show that of all nosocomial infections reported to the National Healthcare Safety Network, 3% were due to VRE [4]. In Europe, Enterococcus spp. was isolated from 9.6% of all nosocomial infections. Furthermore vancomycin-resistance was reported in 10.2% of isolated enterococci (ECDC-Annual-report 2014). The incidence of nosocomial infections caused by VRE is particularly high on intensive care units (ICU) [1, 5]. Risk factors associated with colonization and subsequent development of nosocomial infections due to VRE are severe underlying health conditions such as liver transplantation, neutropenia, diabetes mellitus or renal dysfunction [6–8]. Several studies have shown that VRE bloodstream infections (BSI) are associated with a significantly higher mortality compared to BSI by vancomycin-susceptible *enterococci* (VSE) [9, 10].

VRE infections result in a greater number of invasive procedures, additional antimicrobial therapy, and an extended length of stay, all of which can increase the total hospital costs [8, 11–14]. Hospital costs due to VRE infections such as BSI varied between $9949 and $77,558 in a university-based teaching hospital in the USA (calculated in 2003) [9, 15]. Another study in the USA revealed that attributable costs for surgical site infections (SSI) due to VRE were about $12,766 [16]. In contrast to these findings other investigators suggested that VRE infections and VSE infections do not differ in attributable costs, length of stay (LOS) and mortality [17]. Nevertheless, the Centers for Disease and Control and Prevention (CDC) as well as the German Society for Hygiene and Microbiology (DGHM) have developed infection control measures that aim to prevent VRE transmission [18, 19]. VRE screening in high risk areas and isolation of patients with VRE are therefore well-established in many hospitals despite the costs for those precautions.

At present studies on the economic burden of VRE infection in hospitalized patients have been performed in the USA and other countries but not in Europe, with its highly developed health care system. In the current study we compared hospital costs between patients with VRE infection and patients with VSE infections to determine the costs that are directly attributed to the vancomycin resistance. In addition, we determined factors that may be associated with increased costs of VRE patients in comparison to VSE patients.

## Methods

### Setting

This study was performed in a 1520-bed, tertiary-care university hospital in Hannover, Germany. The hospital provides 146 intensive care unit (ICU) beds with focus on patients that undergo bone marrow and solid organ transplantation and other surgical procedures. There is an average of 59,000 in-patients annually.

### Study design and data collection

A retrospective case-control study of patients with nosocomial infections caused by VRE and VSE admitted between January 2005 and December 2008 was performed. Cases as well as control patients were identified by searching the database of the clinical laboratory of the Institute of Medical Microbiology and Hospital Epidemiology at Hannover Medical School for VRE and VSE. Searching through medical records of patients the following data were recorded: age, gender, length of stay (LOS), duration of mechanical ventilation, whether the patient was on an ICU or non-ICU when the infection occurred, underlying diseases according to the Charlson comorbidity index, and fatal outcome [20].

### Cases

A case-patient was defined as a hospitalized patient developing a nosocomial infections caused by a VRE. Initially, we excluded all VRE-cultures from urine and faeces. For the remaining VRE-positive specimens, by using CDC criteria, we only considered patients with surgical site infection (SSI), blood stream infection (BSI), intraabdominal infections and infections of organs within visceral cavity due to VRE. Infections were considered nosocomially acquired if the onset of symptoms occurred ≥48 h after admission [21]. The date of taking samples for microbiological examination was considered being the date of the onset of symptoms (infection) also.

### Controls and matching criteria

A control-patient was defined just as the above mentioned cases, but suffering from an infection by a VSE instead of VRE. As done before in similar studies by others [22–24], cases were then matched to controls in a ratio of 1:1 using the following criteria: same type of nosocomial infections, age (±10 years), Charlson comorbidity index (±1), admission and discharge within the same year, and “time at risk” defined as in-hospital stay at least as long as that of cases before VRE infection occurred. Cases on ICU where matched to controls on ICU, and cases on non-ICU were matched to controls on non-ICU.

### Costs and reimbursement

Total hospital costs for cases and controls were provided by the financial control department of the facility. Total hospital costs were separated into hospital costs before and after the onset of the infection. We also stratified costs for medical staff, for medical products, and for drugs. The costs attributable to vancomycin resistance were defined as the difference between costs for VRE patients and costs for VSE patients. Individual reimbursement data based on the German diagnosis related groups (G-DRGs) was also provided by the financial control department. Hospital loss per patient was defined as the exceeding amount of costs compared to reimbursement by health insurance companies.

### Microbiological methods

Initially we searched the database of the clinical microbiology laboratory for cultures of both VRE and VSE from blood cultures, wound swabs, wound drainages, intraoperative swabs, as well as gallbladder fluids and ascites. Bacterial cultures were done using standard media. Antibiotic sensitivity profiles were obtained by using the VITEK-2-XL system (bioMerieux, Nuertingen, Germany), and the Merlin MICRONAUT Sprint Dispenser automated broth micro-titer system (Genzyme Virotech, Ruesselsheim, Germany), respectively.

### Statistical analysis

A sample size calculation was not done, as this study was a retrospective approach with a limited number of cases available. For the cases and controls, we calculated for continuous parameters the median and the interquartile range (IQR) and for binary parameters number and percentages. Differences between the groups were tested using the paired Wilcoxon rank sum test for continuous variables, and the paired Fisher’s exact test for binary variables.

Attributable costs and loss due to vancomycin-resistance in enterococci infections were calculated by matched pairs as the difference between VRE cases and VSE controls and tested by paired Wilcoxon rank sum test for paired samples as done before [25]. To identify independent risk factors for the costs, a univariable and a multivariable analysis was performed using generalized estimating equation (GEE). To achieve normal distribution, costs were log transformed. The regression coefficients were converted to the measures of effect using an exponential transformation and referred to as the multiplicative effect (ME) of patient characteristics and hospital events. The univariable and multivariable analysis calculate GEE models that take the correlation of matching between cases and control in account by applying an exchangeable correlation structure.

All parameters with *p* < 0.05 in the univariable GEE-models were considered in the multiple analysis and included in a full model. From this model, parameters with the smallest Chi-Square value and *p* ≥ 0.05 were excluded stepwise until the *p*-value of all parameters included in the model were <0.05. Data were analysed by SAS 9.1. A value of *p* < 0.05 was considered significant.

## Results

The initial search of the microbiological database provided 504 patients with VRE. 403 patients were excluded because they had no signs of infection, or infection was not nosocomial. 101 cases were included as cases. VSE were detected in 5502 patients, of whom 936 patients had nosocomial infections. Matching was possible for 42 cases, thereof 41 were affected by Vancomycin-resistant *E. faecium* and one by Vancomycin-resistant *E. faecalis*. Those were then compared to 42 controls with VSE infection, thereof 24 infected by *E. faecium* and 18 infected by *E. faecalis*.

### Patients´ characteristics

There were no significant differences between the two groups with respect to age, gender, fatality rate, overall LOS, duration of mechanical ventilation and Charlson comorbidity index (Table 1).

**Table 1:** Characteristics of cases with nosocomial VRE infection and controls with nosocomial VSE infection

Characteristics	VRE infection (*n* = 42)	VSE infection (*n* = 42)	*p-*value ª
Clinical criteria			
Age (years)^b^	50 (32–65)	58 (43–67)	0.190
Male	23 (54.8%)	23 (54.8%)	1.000
No. of patients on intensive care unit at time of onset of infection^b^	39 (92.9%)	39 (92.9%)	1.000
Mechanical ventilation (h)	169 (5–471)	25 (3–302)	0.294
Charlson comorbidity index^b^	3 (1–4)	3 (1–4)	0.906
Cardiac insufficiency	4 (9.5%)	6 (14.3%)	0.738
Peripheral vascular disease	1 (2.4%)	0 (0%)	1.000
Cerebrovascular disease	4 (9.5%)	0 (0%)	0.116
Chronic pulmonary disease	1 (2.4%)	3 (7.1%)	0.616
Mild liver disease	10 (23.8%)	8 (19%)	0.791
Hemi- or paraplegia	2 (4.8%)	1 (2.4%)	1.000
Kidney disease	9 (21.4%)	7 (16.7%)	0.782
Malignant tumour	12 (28.6%)	12 (28.6%)	1.000
Severe liver disease	3 (7.1%)	0 (0%)	0.241
Metastasized malignant tumour	1 (2.4%)	1 (2.4%)	1.000
Myocardial infarction	0 (0%)	2 (4.8%)	0.494
Outcome			
LOS (days) after diagnosis of infection	33 (13–63)	27 (14–35)	0.122
LOS (days) before diagnosis of infection	16 (10–26)	15 (9–25)	0.730
Overall LOS (days) in the hospital	54 (35–80)	45 (27–63)	0.139
Death	14 (33.3%)	11 (26.2%)	0.634

LOS, length of stay; Values are numbers (%) or medians (interquartile range)

ªWilcoxon rank sum test for continuous variables and Fisher’s exact test for binary variables

^b^Additional matching criteria were admission and discharge within the same year, and “time at risk” defined as a length of stay at least as long as that of cases before the onset of VRE infection

### Costs and loss

The total hospital costs for a patient with VRE infection were significantly higher than those for patients with VSE infection (Table 2). Hospital costs directly attributed to vancomycin-resistance were calculated by the difference in costs of matched pairs and summed up to EUR 13,157. There was no significant difference in the costs between the two groups before the onset of the infection. In contrast, costs after onset of infection were significantly higher in cases compared to controls (Table 2). The hospital loss per patient with VRE infection was somewhat higher compared to VSE infections, but did not reach statistical significance.

**Table 2:** Costs and reimbursement for nosocomial VRE and VSE infections

	VRE infection (*n* = 42)	VSE infection (*n* = 42)	*p-*value^a^
Costs per patient (EUR)	57,675 (34,253 to 124,079)	38,344 (20,872 to 76,353)	0.030
Costs per patient before the onset of the infection (EUR)	17,893 (10,664 to 32,191)	16,600 (7373 to 24,723)	0.386
Costs per patient after the onset of the infection (EUR)	37,971 (17,786 to 80,662)	23,025 (10,322 to 40,565)	0.049
Reimbursement per patient (EUR)	40,084 (20,128 to 99,501)	26,116 (10,898 to 57,139)	0.032
Loss per patient (EUR)	14,003 (1974 to 32,743)	6883 (3123 to 20,511)	0.227
Median costs attributable to vancomycin-resistance in enterococci infection per patient (EUR)^b^	13,157(−16,420 to 74,593)		0.036

Values are medians (interquartile range)

^a^Paired Wilcoxon rank sum test for paired sample

^b^Differences between case and control

Detailed analysis of costs between VRE and VSE patients after the onset of infection showed the highest significant differences for pharmacy (EUR 6030 vs. EUR 2801; *p* = 0.008) followed by nursing staff (EUR 8956 vs. EUR 4621; *p* = 0.032), medical products (EUR 3312 vs. EUR 1838; *p* = 0.020), and assistant medical technicians (EUR 3766 vs. EUR 2474; *p* = 0.023).

Multivariate analysis revealed independent predictors for increased hospital costs in patients with enterococci infections. The impact on the costs by these independent predictors is given as the multiplicative effect (ME) (Table 3). Significant independent predictors were, mechanical ventilation >500 h (ME = 2.91), severe liver diseases (ME = 1.94), peripheral vascular disease (ME = 1.58), vancomycin-resistance (ME = 1.37), and myocardial infarction (ME = 1.34).

**Table 3:** Multivariable analysis of the impact of clinical characteristics on hospital costs for *enterococci* infections (significant results with *p* < 0.05 only)

Clinical Characteristics	ME (CI95%)		*p-*value
Mechanical ventilation >500 h	2.91	(2.17–3.91)	<0.001
Severe liver disease	1.94	(1.03–3.66)	0.041
Peripheral vascular disease	1.58	(1.23–2.03)	<0.001
Vancomycin-resistance	1.37	(1.02–1.83)	0.034
Myocardial infarction	1.34	(1.04–1.71)	0.021
Age > 60 years	0.72	(0.53–0.98)	0.035

ME, multiplicative effect; CI95%, 95% Confidence interval

In contrast age > 60 years was a predictor for decreased hospital costs (ME = 0.72).

## Discussion

Increasing antimicrobial resistance is a growing problem for patients as well as for health care systems with respect to continuously growing hospital costs. Accurate estimates of the financial outcome associated with nosocomial infections caused by multidrug resistant organisms are still rare but they remain important for evaluating the cost effectiveness of prevention strategies to reduce transmission.

Attributable hospital costs for vancomycin-resistance per patient were EUR 13,157. Other authors calculated attributable hospital costs for vancomycin-resistance ranging from $ 1546 to $ 77,558 [9, 14–17, 26]. Three studies looked at blood stream infections (BSI) [9, 14, 15], one study looked at wound infections [16], one study looked at wound infections, urinary tract infections, and blood stream infections (BSI) [17], and one study looked at wound infection, urinary tract infections, blood stream infections (BSI) and intraabdominal infections [26]. In these studies, the total hospital costs ranged from $ 33,224 to $ 124,257 for a patient with VRE, from $ 20,895 to $ 56,707 for a patient with VSE, or from $ 8192 und $ 18,863 for a patient with neither VRE nor VSE, respectively [9, 14, 15, 17]. The wide range of costs are probably caused by the difference in the study design, differences in matching criteria, the size of the study population, and differences in adjustment for confounders. For example, Pelz et al. compared costs between cases and unmatched controls [17]. Song et al., even though so they matched controls for age, year of admission, morbidity and length of stay prior to VRE infection, they did not consider other factors that affect costs such as overall length of stay or stay on the ICU [9]. However although costs for VRE infections vary considerably between published studies, the key message remains: vancomycin-resistance in *enterococci* is associated with increased costs when patients suffer from infections due to VRE.

Multivariable analysis in this study showed that a VRE-infection was independently associated with a 1.4-fold increase in total costs per patient. This confirms previous findings by Kaye et al., who found also a 1.4 fold increase in total costs per patient [16]. Other studies found similar multiplicative effects (ME) ranging from 1.2 to 1.6 [9, 26]. Carmeli et al., who looked at four different VRE infections, found a ME of 1.2 for blood stream infections (BSI), a ME of 1.3 for urinary tract infection, a ME of 1.5 for wound infection, and a ME 1.6 for intraabdomial infections [26]. We could also show that age > 60 years was associated with lower (!) costs. These findings are somehow contrary to the data of Webb et al., who found a positive correlation of age (>50 years) and increased costs [27]. This may at least in part be explained by mortality in our older patient population. An early fatal outcome will diminish the patient’s length of stay and, by this attributive costs. A similar phenomenon may apply to patients with severe underlying diseases. A high Charlson Comorbidity Index may lead to increased and in particular early mortality, which in turn will then reduce the overall costs.

Previous studies did not differentiate between costs before and after VRE infection, but calculated costs for the total length of stay instead. Here we looked at costs prior to VRE infection and after the patient had developed VRE infection. Our study clearly shows, that it is indeed the VRE infection itself that drives costs, as the costs between patients with VRE and with VSE did not differ before the infection with *enterococci* occurred (EUR 17,893 vs. EUR 16,600; *p* = 0.386), but only after they had developed the infection (EUR 37,971 vs. EUR 23,025; *p* = 0.049).

One may argue that the increased costs of VRE patients could in fact be due to comorbidities rather than the infection itself. Patients with VRE infections often show an increased morbidity, which influences clinical outcome and might indirectly affect costs [10, 27–30]. Several surrogate parameters such as the overall length of stay or various comorbidity scores are being used as a measure of morbidity [28]. In a study by Webb et al., differences between VRE and VSE patients became apparent when the authors stratified for a case-mix-index ≤1, whereas matching patients with a case-mix-index >1 did not generate significant differences in costs between the two groups [27]. Thus to avoid confounding, we applied the Charlson comorbidity index for matching an appropriate control group. We also matched for the time at risk to apply a second surrogate parameter for morbidity. Both surrogate parameters have been used as matching parameters in previous case-control studies looking at costs due to nosocomial infections [24, 31].

It is noteworthy that in contrast to other matched case-control studies comparing costs between VRE and VSE patients that found significant differences in mortality between the two groups [9, 29], in this study we did not detect significant difference in mortality between cases and controls (33% vs 26%, *p* = 0.634). The meta-analysis by Salgado et al. showed an increased mortality of VRE patients in comparison to VSE patients in most studies [10]. Only few studies, all of which had a small study population (6 to 46 VRE cases) did not show a difference in mortality. The small study size might be one reason why we failed to detect a significant difference in mortality between cases and controls in the present study. Beside the size of the study population, the quality of the matching criteria will also greatly influence the mortality for cases compared to controls, because the Charlson comorbidity Index was particularly developed to estimate the risk of death from comorbid disease [20]. In the present study the Charlson comorbidity Index (CCI) was identical between cases and controls (CCI = 3), which might provide another explanation for equal mortality between cases and controls.

Detailed analysis of costs between patients with VRE and VSE showed significant differences for pharmaceuticals, nursing staff, medical products, and for assistant medical technicians. The most significant differences turned out to be for pharmaceuticals (EUR 6030 vs. EUR 2801; *p* = 0.008). This might be due to treatment with second and third line antibiotics such as linezolid or quinupristin/dalfopristin. Similar stratifications are scarce in other studies looking at costs of VRE infections. Geahart et al. did not find differences when stratifying for costs for pharmaceuticals, but did find differences for the laboratory costs [29]. Butler et al. found significant differences in costs for room and board, pharmaceuticals, laboratory and radiology [15]. Differences in the costs are most probably due to the prolonged length of hospital stay after the onset of VRE infection. VRE cases and VSE controls were matched for the time at risk (before infection), but VRE cases presented with a significant longer hospital stay thereafter. This prolonged time largely attributes to the cost calculation including the use of medical products and technical staff.

Our study has some limitations. First, all patients included in the analysis stayed at the Hannover Medical School in Germany. Thus results of this single-centre study may not apply to other institutions. Second, the small sample size of 84 patients for cases and controls each limits this study. However, due to the application of strict matching criteria, we were not able to further increase the study population. Although we did match for the type of infection, we did not differentiate between types of infection in terms of cost calculation and calculation of the multiplicative effect due to the limited number of case patients as it has done by others before [32, 33]. On the other hand, fewer matching criteria would have led to less power of statistical analysis, and we consider our type of matching as the main strength of this study. Particularly using “time-at-risk” for matching cases and controls limited the sample size. But including “time-at-risk” was important because Cosgrove et al. showed a significant correlation between time-at-risk and financial outcome [28]. Third, we did not evaluate the financial impact of VRE infection from the perspective of the patient and the society, such as lost wages, or the impact on life-time-quality. Finally, recent evidence suggests that even matching on infection onset still provides slight overestimates of costs attributable to infection [34, 35], but this should only have had a minor impact on the overall results.

## Conclusion

To our knowledge this retrospective case-control study of infections with VRE and VSE provides the first data of its kind in Europe. We demonstrated that attributable costs for vancomycin-resistance in *enterococci* in patients who develop infections with this pathogen are considerable, and that vancomycin-resistance is an independent predictor for the overall increase of hospital costs. Infections with VRE not only increased costs for the health care system, but, due to the reimbursement policy, even generates loss for the individual hospital.

These results emphasise the importance of strategies for the prevention of VRE spread in the hospital.

## Abbreviations

BSI: Bloodstream infection
CCI: Charlson comorbidity Index
CDC: Centers for Disease Control and Prevention
DGHM: German Society for Hygiene and Microbiology
ECDE: European Centers for Disease Control and Prevention
G-DRGs: German diagnosis related groups
GEE: Generalized estimating equation
ICU: Intensive care unit
IQR: Interquartile range
LOS: Length of stay
ME: Multiplicative effect
NI: Nosocomial infection
SSI: Surgical site infection
VRE: Vancomycin-resistant *enterococci*
VSE: Vancomycin-susceptible *enterococci*
